# Enhanced Cytotoxic Effects of Docetaxel‐Loaded Solid Lipid Nanoparticles (SLN‐DTX) on Gastric Adenocarcinoma In Vitro

**DOI:** 10.1002/jbt.70456

**Published:** 2025-08-21

**Authors:** Laís Vaz‐Costa, Marina Arantes Radicchi, Caterynne Melo Kauffmann, Guilherme Sirimarco de Souza Silveira Tonelli, Igor Oliveira Santos, Kelly Grace Magalhães, Sônia Nair Báo

**Affiliations:** ^1^ Graduate Program in Biological Sciences (Molecular Biology), Department of Cell Biology, Institute of Biological Sciences University of Brasilia Brasília Brazil; ^2^ Laboratory of Microscopy and Microanalysis, Institute of Biological Sciences University of Brasilia Brasília Brazil; ^3^ Laboratory of Phytopathology, Institute of Biological Sciences University of Brasília Brasília Brazil; ^4^ Laboratory of Immunology and Inflammation, Department of Cell Biology University of Brasilia Brasília Brazil

**Keywords:** AGS, antitumor effect, cell proliferation, drug carrier, inflammation

## Abstract

Gastric cancer is one of the leading causes of mortality worldwide. Despite recent advances in cancer therapy, there has been little change in cure and survival rates in patients suffering from gastric cancer. The use of conventional chemotherapeutic agents such as docetaxel is limited by some disadvantages such as alopecia and hematotoxicity. This study focused on the antitumor effects of solid lipid nanoparticles loaded with docetaxel (SLN‐DTX) on gastric adenocarcinoma cells. SLN‐DTX showed cytotoxicity against cancer cells (AGS), demonstrating an IC50 value lower than the concentration of free DTX after 24 h of treatment, evidencing the efficiency of nanoparticles. Morphological analysis revealed structural alterations, including cell rounding and reduced cytoplasmic projections posttreatment. SLN‐DTX and DTX induced damage to microtubules, binding proteins, and core fragmentation, impairing cell adhesion and proliferation. They also showed changes in analyses involving cellular organelles (mitochondria and lysosomes) and cellular metabolism. SLN‐BLANK did not show significant toxicity in the AGS tumor lineage in any assays, behaving similarly to the untreated control. Association of docetaxel with solid lipid nanoparticles (SLN‐DTX) demonstrates significant efficiency in inducing cytotoxic effects in gastric adenocarcinoma cells, since this formulation exhibited potent antitumor activity. Treatment with nanostructured docetaxel caused morphological and metabolic changes in addition to altering the release of molecules relevant to tumor progression. These findings support the potential use of SLN‐DTX as a promising drug delivery system, offering an innovative and effective approach for gastric cancer treatment while potentially reducing the side effects associated with conventional therapies.

## Graphical abstract

Using nanotechnology as a tool for cancer treatment, the solid lipid nanoparticle of docetaxel (SLN‐DTX) promotes toxicity in human gastric adenocarcinoma cells (AGS), reducing viability and proliferation, morphological, and metabolic changes, in addition to immune response alterations.

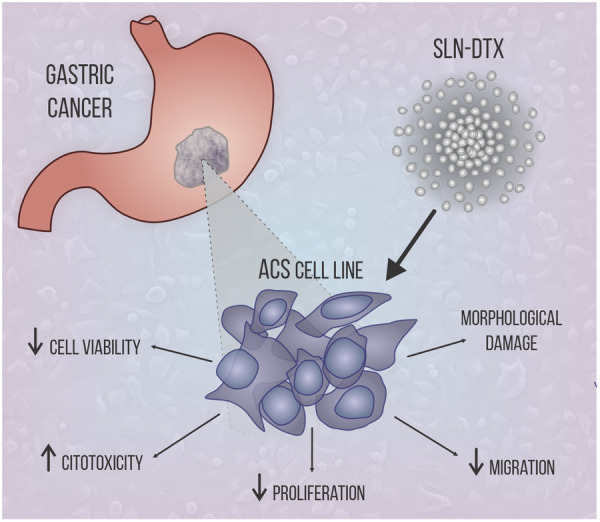

## 1 Introduction

Gastric cancer (GC) appears as one of the most common cancers and is the third leading cause of cancer‐related death worldwide, with more than one million patients diagnosed with GC globally every year [1, 2]. Currently, the primary therapy for patients with GC is surgery and systemic chemotherapy; radiotherapy, immunotherapy, and targeted therapy are gradually being used [3, 4, 5]. However, the 5‐year survival rate of patients undergoing conventional treatments is still unsatisfactory [1, 5].

Docetaxel (DTX) is one of the main chemotherapeutic agents used in the first‐line treatment of breast, ovarian, bladder, prostate, gastric, and non‐small cell lung carcinomas [6]. It was approved by the Food and Drug Administration (FDA) and is part of the pharmacological group called “taxanes,” which are derived from a natural substance found in the bark of the yew tree, *Taxus baccata*, a tree that produces toxic substances (taxins) and also medicinal ones (taxol) [7, 8]. Its commercial formulation containing docetaxel, Taxotere©, employs polysorbate 80, a non‐ionic surfactant related to hypersensitivity relationships [9]. Treatment with taxanes is known to cause a series of adverse effects related to the dose used, ranging from allergic reactions and dermatological toxicity to hematological changes [6].

Considering the points raised, it is necessary to optimize therapy for GC, either through the search for new molecules or through pharmaceutical technology, such as the use of nanoparticles, which could allow greater treatment efficiency. The limitations of the anticancer drug Taxotere® challenge researchers in the search for the insertion of the active ingredient docetaxel (DTX) into nanocarriers. DTX in association with Nanoparticles (NPs) brings advantages such as low toxicity, controlled drug release, stability and protection as well as high drug loading. The non‐use of organic solvents in the preparation, biodegradability, biocompatibility, and ability to incorporate hydrophilic and hydrophobic compounds can also be mentioned [10, 11]. Therefore, the objective of this study was to evaluate the efficiency of Solid Lipid Nanoparticles containing Docetaxel (SLN‐DTX) in the in vitro treatment of gastric adenocarcinoma.

## 2 Materials and Methods

### 2.1 Materials

Docetaxel, Pluronic F127, Span 80, Compritol ATO 888, DAPI, and Formvar were purchased from Sigma‐Aldrich, USA. Human gastric adenocarcinoma (AGS) cells were acquired from the Rio de Janeiro Cell Bank. Dulbecco′s modified Eagle′s medium (DMEM), fetal bovine serum (FBS), penicillin, and streptomycin were purchased from Gibco, Invitrogen, USA. MTT (3‐[4,5‐dimethylthiazol‐2‐yl]−2,5 diphenyltetrazolium bromide), Cell Trace CFSE Cell Proliferation Kit Protocol Flow Cytometric, and Prolong Gold Antifade were purchased from Thermo Fisher, USA. Glutaraldehyde, Epon resin, osmium tetroxide, and uranyl acetate were purchased from Polysciences, USA.

### 2.2 Cell Maintenance

The AGS (Gastric Adenocarcinoma), and HNTMC (fibroblasts isolated from human dental pulp) cells were cultured in DMEM medium with 1% streptomycin and penicillin solution, and 1% FBS, when necessary, and maintained in a monitored environment at 37°C with 5% CO_2_.

### 2.3 Docetaxel Solid Lipid Nanoparticle Formulation

Solid lipid nanoparticles were formulated by high energy method, adapted from da Rocha et al. (2020) [12], where a lipid phase composed of Compritol® 888 ATO, Pluronic F127, and Span 80 was heated at 80°C, then PBS was added to the formulation, and the sample (SLN‐Blank) was dispersed by high energy shaking using the Ultra Turrax T25 (IKA). For SLN‐DTX, 1 mg/mL docetaxel was added in the lipid phase, and commercial docetaxel was diluted in ethanol at the same concentration as control (DTX). For the evaluation of cellular internalization, docetaxel was replaced by 30 mM aluminum‐chloro‐phthalocyanine, named SLN‐FTALO.

### 2.4 Cell Viability Assay

Cells were treated with SLN‐DTX to evaluate cell viability and obtain IC50. 5 × 10^3^ cells were deposited in a 96‐well plate. After 24 h, cells were treated with docetaxel concentrations up to 100 μg/mL. In the case of SLN‐Blank, volumes equivalent to those of SLN‐DTX were used. Treatment times were 24, 48, and 72 h, after which the cell viability assay was performed with 3‐(4,5‐dimethylthiazol‐2‐yl) bromide −2,5‐diphenyltetrazolium (MTT) as instructed by the manufacturer. After quantification, the data were applied to dose–response equations in GraphPad Prism (GraphPad Software Inc.) to obtain the IC50 for each treatment.

### 2.5 Real‐Time Cell Analysis (RTCA)

To evaluate cell viability in real time, the Real‐Time Cell Analysis system xCELLigence (ACEA) was used. After plating and 24 h of adaptation, the equivalent of 10 μg/mL of docetaxel per well was added, and the behavior of the cells was constantly evaluated every 30 min for 120 h in a controlled environment at 37°C and 5% CO_2_.

### 2.6 Electron Microscopy

To evaluate cell morphology after treatment, AGS cells were treated with 10 μg/mL NLSDTX for 24 h. Treated cells were fixed overnight with Karnovsky (2% paraformaldehyde, 2% glutaraldehyde in 0.1 M sodium cacodylate buffer, pH 7.2) and then washed with sodium cacodylate buffer. The samples were post‐fixed with 1% osmium tetroxide and 0.8% potassium ferrocyanide, then dehydrated with increasing concentrations of acetone (30%–100%).

For transmission electron microscopy, the samples were embedded in Epon resin and sectioned into ultrathin sections to be analyzed using the JEOL 1011 transmission electron microscope.

For scanning electron microscopy, the samples were contrasted with 0.5% uranyl acetate; after dehydration, they were subjected to critical point drying (CPD 030, BALZERS, USA) and metalized (SCD 500, LEICA, Germany) to be viewed under the scanning electron microscope (JEOL, JSM‐700 1‐F, Japan).

### 2.7 Cell Morphology Under Confocal Microscopy

To evaluate the possible interaction of SLN‐DTX with the cytoskeleton, AGS cells were treated with 10 μg/mL SLN‐DTX for 24 h, then fixed with 3.7% formaldehyde for 15 min; 0.1% Triton X‐100 in PBS was used to permeate the cells. To avoid nonspecific binding, samples were blocked with 1% skimmed milk, 2.5% bovine serum albumin (BSA), and 8% fetal bovine serum (FCS) in PBS—pH 7.4.

For labeling, cells were incubated at 4°C overnight with the anti‐β‐tubulin primary antibody (1:500), followed by incubation in the IgG Alexa‐488 secondary antibody (5 μg/mL) for 1 h. Subsequently, the coverslips were incubated for 7 min with DAPI (300 nM) for DNA labeling. The slides were mounted and analyzed using a Confocal Laser Scanning Microscope (LEICA TCS SP5).

### 2.8 SLN‐FTALO Internalization

Flow cytometric analysis of cells treated with SLN‐FTALO (30 μM) allows the identification and quantification of subpopulations of cells that have internalized and/or have nanoparticles present in their membrane and begin to present fluorescence inside and on their membrane. The incubation times with NLS‐AIPc were: 0 min, 30 min, 1 h, 3 h, 6 h, 9 h, 15 h, 18 h, 21 h, and 24 h. The analysis was performed on a FACSCalibur cytometer with the aid of CellQuest‐Pro software. The analyses were performed from three independent experiments.

### 2.9 Endocytosis Pathways

The endocytosis pathway by which SLN‐FTALO is internalized by AGS cells was evaluated. The inhibitors used were: Filipin (1 μg/mL) and Nystatin (20 μg/mL), which block caveolin‐mediated endocytosis; Amiloride (0.2 mM) and Cytochalasin D (1 μM), which inhibit macropinocytosis; and phenylarsine oxide (0.2 μM), which inhibits clathrin‐mediated pathways. Afterward, the cells received treatment with SLN‐FTALO (30 μM) for 2 h. Cells were analyzed by FACSCalibur flow cytometry, and 10,000 events were counted per sample. The results were processed by the FlowJO program.

### 2.10 Mitochondrial Density

For quantification and visualization of mitochondria, AGS cells were treated with 10 μg/mL SLN‐DTX, 150 nM MitoTracker Green FM was used as a fluorescent marker. Per sample 10,000 events were analyzed by flow cytometry (FACS Calibur), and the data were processed using FlowJo vX 0.7 software.

Confocal fluorescence microscopy was used to obtain images of the distribution of mitochondria in AGS cells treated with 10 μg/mL SLN‐DTX after 24 h. Cells were fixed with 3.7% formaldehyde for 15 min and permeabilized with 0.1% Triton X‐100 in PBS for 20 min. Then the cells were incubated with MitoTracker® Green at a concentration of 150 mM for 30 min. Afterward, the coverslips were incubated for 7 min with DAPI (300 nM) to label DNA. After preparing the samples, the slides were analyzed using a confocal laser scanning microscope (LEICA TCS SP5).

### 2.11 Cell Proliferation Assay

To evaluate the cell proliferation rate, AGS cells treated with 10 μg/mL SLN‐DTX for 48 h were exposed to 5 μM CFSE (eBioscience) diluted in PBS for 15 min at 37°C. Subsequently, the cells were analyzed in the flow cytometer using the green fluorescence channel (FL‐1), and data were analyzed with FlowJo vX. CFSE‐labeled AGS cells were exposed to 5 μM colchicine for 24 h as a nonproliferation control.

### 2.12 Cell Death Pathway Assay

Flow cytometry was used to determine the cell death pathway. AGS cells were treated with 10 μg/mL SLN‐DTX for 24 h and then labeled with annexin V and 2 g/mL propidium iodide. For apoptosis control, the cells were treated with 10 mM hydrogen peroxide. As necrosis control, the cells were subjected to 90°C heating. Quantitative cell analyses were performed in FACSCalibur, and data were processed in CellQuest‐Pro.

### 2.13 Reactive Oxygen Species Assay

To evaluate the oxidative stress caused by the treatment, AGS cells were treated with 10 μgmL SLN‐DTX for 24 h. Hydrogen peroxide was used as a positive control to produce reactive oxygen species (ROS). Treated cells were incubated at 37°C for 30 min with 5 μM CellROX. ROS production was quantified by flow cytometry using FACSCalibur, and data were analyzed using FlowJo xV 0.7 software.

### 2.14 Determination of Intracellular Ca^2+^ Level

To determine the intracellular Ca^2+^ level, 3 × 10⁵ AGS cells were plated in 12‐well plates. After 24 h and 48 h of treatment with 10 µg/mL of SLN‐DTX, DTX, SLN‐BLANK, and culture medium (control), cells were trypsinized and washed with PBS. The cells were then exposed to 2 µL of Fluo‐4/AM for 30 min at room temperature and protected from light. The intracellular Ca^2+^ level was analyzed using the FL1 channel on the FACSCalibur cytometer (Becton Dickinson).

### 2.15 Membrane Potential

Rhodamine 123 was used on AGS cells treated with 10 μg/mL SLN‐DTX to measure mitochondrial membrane potential for 24 h. The cells were incubated in the probe for 15 min protected from light at room temperature, then washed and analyzed on the FACSCalibur (Becton Dickinson) flow cytometer. Membrane potential was evaluated by quantifying fluorescence in 10000 events in triplicate, and data were processed using FlowJo® vX 0.7 software.

### 2.16 Lipid Body Biogenesis Assay

For quantification of lipid bodies, SLN‐DTX treated AGS cells (10 μg/mL) were incubated with Bodipy 493/503 (Life Technologies) for 30 min at 4°C protected from light, afterward analyzed by flow cytometry (FACSCalibur. Histograms and median fluorescence intensity (MFI) were carried out using FlowJo® vX 0.7 software.

### 2.17 Cytokine Quantification

Cytokine levels were measured using commercial ELISA tests according to the manufacturers′ recommendations. The pro‐inflammatory cytokines TNF‐α and IL‐6 and nitric oxide were measured in the supernatant of AGS cells treated with 10 μg/mL of SLN‐DTX. Quantification was performed by colorimetry at 450 nm with SpectraMax.

### 2.18 Wound Healing Assay

AGS cells were cultured and then a scratch was made with a tip in the well. The cells were then treated with 10 μg/mL SLN‐DTX and photographed after 24 and 48 h; colchicine was used as a proliferation control.

### 2.19 Statistics

Data analysis was performed with the support of GraphPad Prism software; images were processed with Corel DRAW 2019, Corel PHOTO‐PAINT 2019, and ImageJ software. Statistics were performed with the removal of outliers by the Grubbs test, and then normality was assessed by the Shapiro‐Wilk test. Comparison between groups was performed through ANOVA with Tukey′s posttest.

## 3 Results

### 3.1 SLN‐DTX Promotes Toxicity in Gastric Adenocarcinoma Cells

**FIGURE 1:**
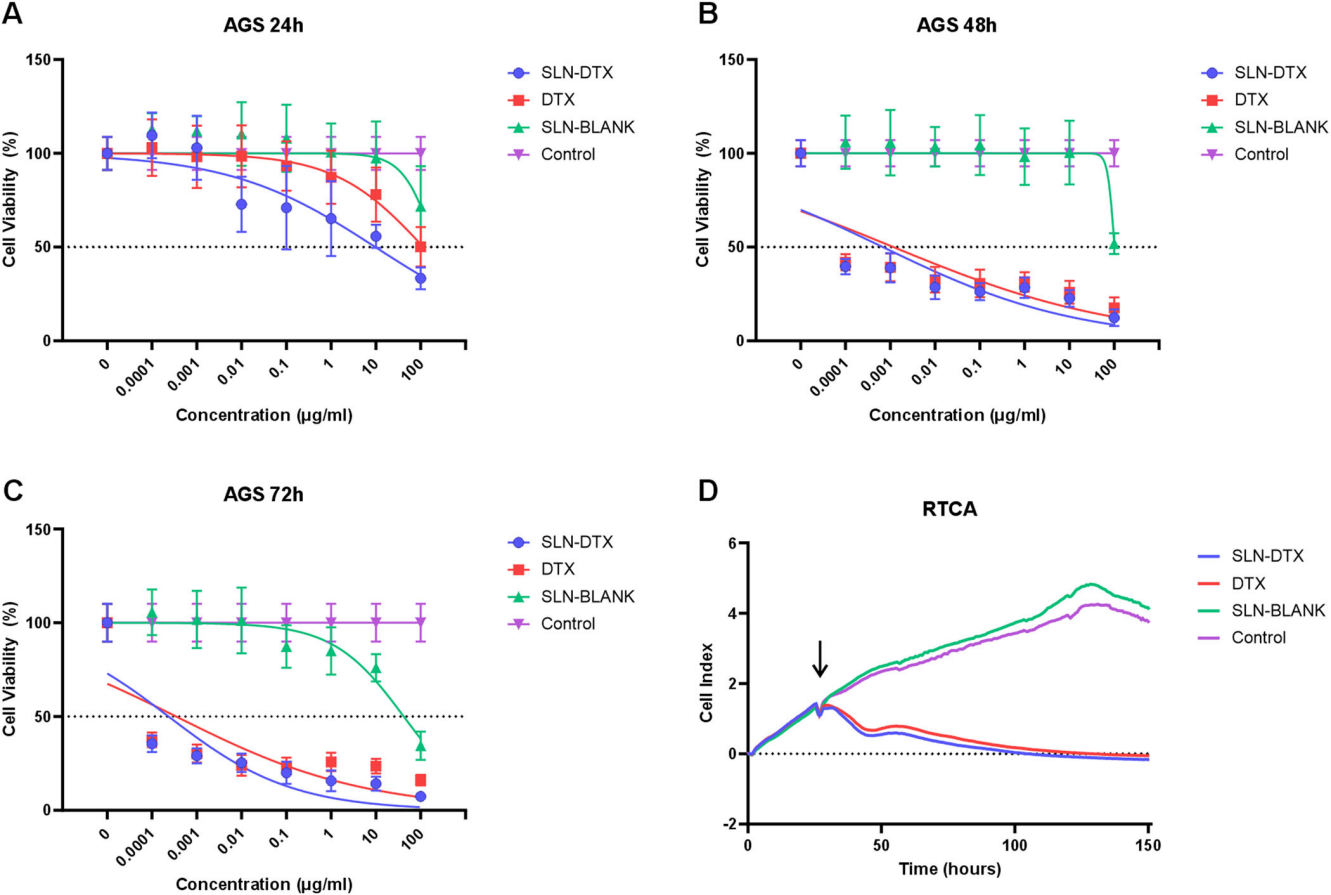
Evaluation of cell viability of SLN‐DTX‐treated AGS cells. Cell viability concerning different concentrations of docetaxel after 24 h (A), 48 h (B), and 72 h (C) of treatment. (D) shows the cell index obtained by Real‐Time Cell Analysis; the arrow indicates the time at which the treatment was added to the cells.

**FIGURE 2:**
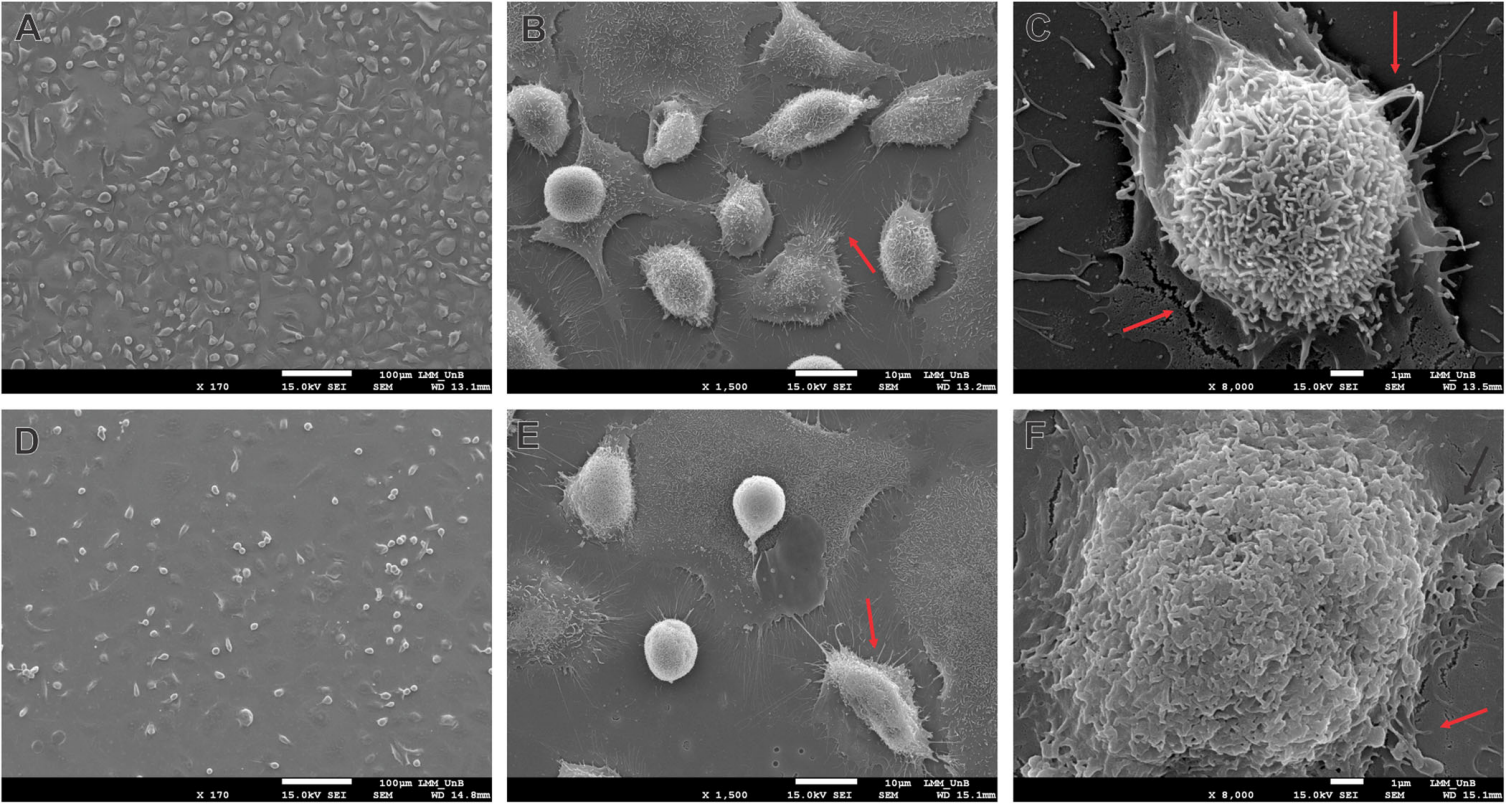
AGS cells under scanning electron microscopy. A, B, and C show micrographs of untreated cells as controls, and D, E, and F show cells treated with SLN‐DTX. Red arrows indicate cytoplasmic extensions.

Human gastric adenocarcinoma (AGS) cells are used as an experimental model for in vitro evaluations of the efficacy of chemotherapy. The use of SLN‐DTX aims to enhance the antineoplastic action of docetaxel. Figure 1 shows the variations in the viability of docetaxel concentrations in three different treatment times, namely 24 h (A), 48 h (B), and 72 h (C). The IC50 obtained for the 24‐h treatment was 9.727 μg/mL for SLN‐DTX and 115.6 μg/mL for DTX. Additional viability essays in nontumor cell lines are shown in Supporting Information S1: Figure 1. Supporting Information S1: Figure 2 presents multiple statistical comparisons for the MTT and RTCA assays.

The evaluation of the cell index of AGS treated with 10 μg/mL of SLN‐DTX is shown in Figure 1D, where the arrow indicates the moment when the treatment was added to the wells, SLN‐DTX and DTX presented similar profiles, but with a reduction in the nanostructured treatment. The efficiency of treatments with DTX in nanostructured vehicles was observed by da Rocha and co‐workers (2020) [12], where the same SLN‐DTX was able to increase the efficiency in reducing the viability in human and murine breast cancer cells (MCF‐7 and 4T1).

Using scanning electron microscopy, it is possible to observe a significant reduction in the number of cells after treatment with 10 μg/mL of SLN‐DTX when compared to the untreated control (Figure 2A,D). With the treatment, fewer cells with an elongated phenotype of greater adherence were observed, with rounded cells more frequent, and uniformly distributed on the surface (Figure 2A,D). The cells presented fewer cytoplasmic extensions, indicated by the red arrows (Figure 4B,E); in addition, their surface presented a different organization when compared to the cells without treatment (Figure 4C,F).

**FIGURE 3:**
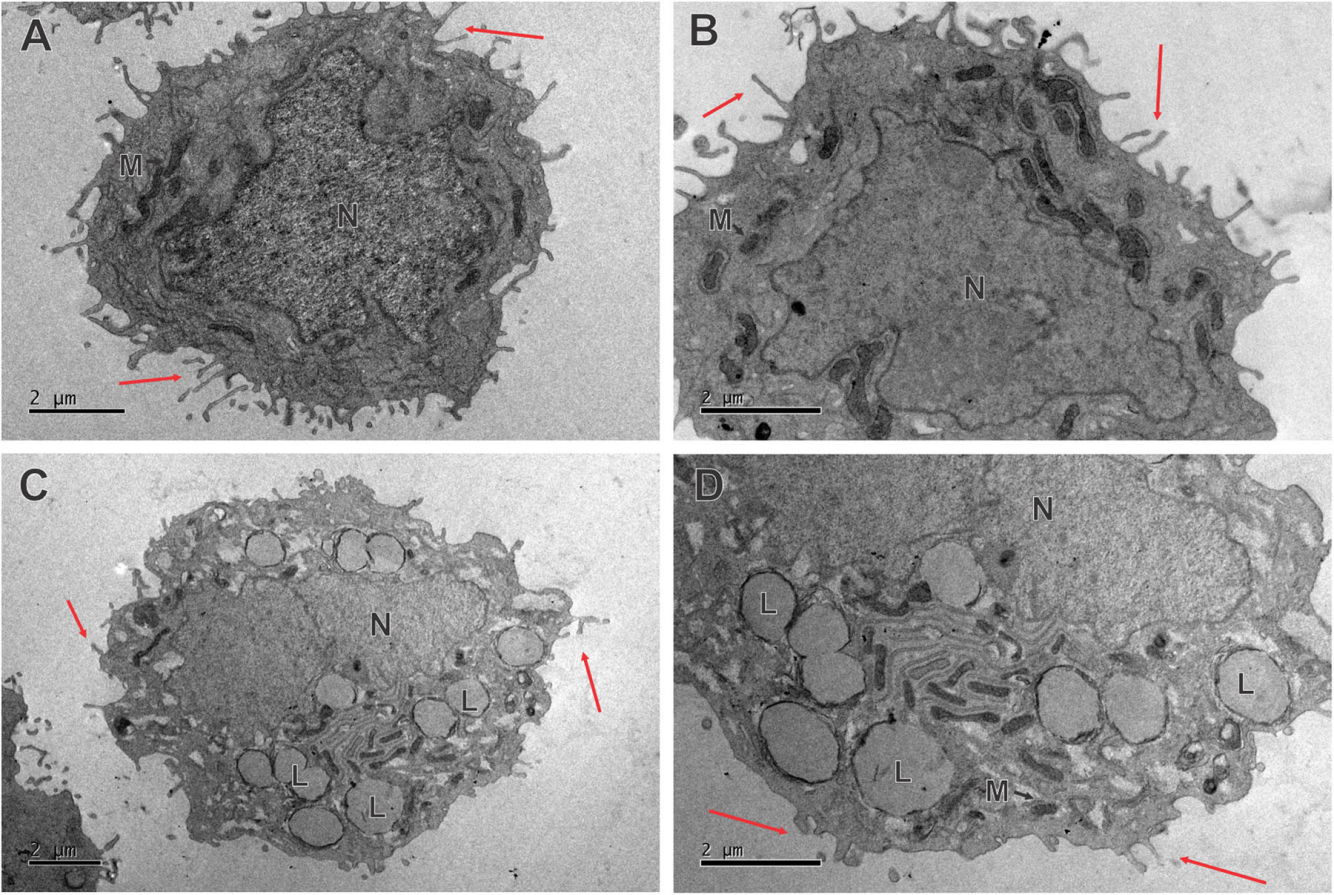
Transmission electron microscopy of SLN‐DTX‐treated AGS cells. A and B present untreated cells, while C and D show SLN‐DTX‐treated cells. Red arrows stand for cytoplasmic processes, N for nucleus, M for mitochondria, and L for lysosomal vesicles.

Corroborating what was observed in Figure 2, there was also a reduction in cytoplasmic projections (red arrows) in transmission electron microscopy (Figure 3). Compared to the control, treatment with SLN‐DTX (Figure 3C,D) altered the morphology of AGS cells, where an increased number of lysosomal vesicles and changes in the organization of the nucleus were observed. In addition to reducing viability, SLN‐DTX prevented wound healing of AGS cells at 24 and 48 h (Supporting Information S1: Figure 3), indicating that the treatment reduces cell viability, alters morphology, and impairs cell recovery after stress.

**FIGURE 4:**
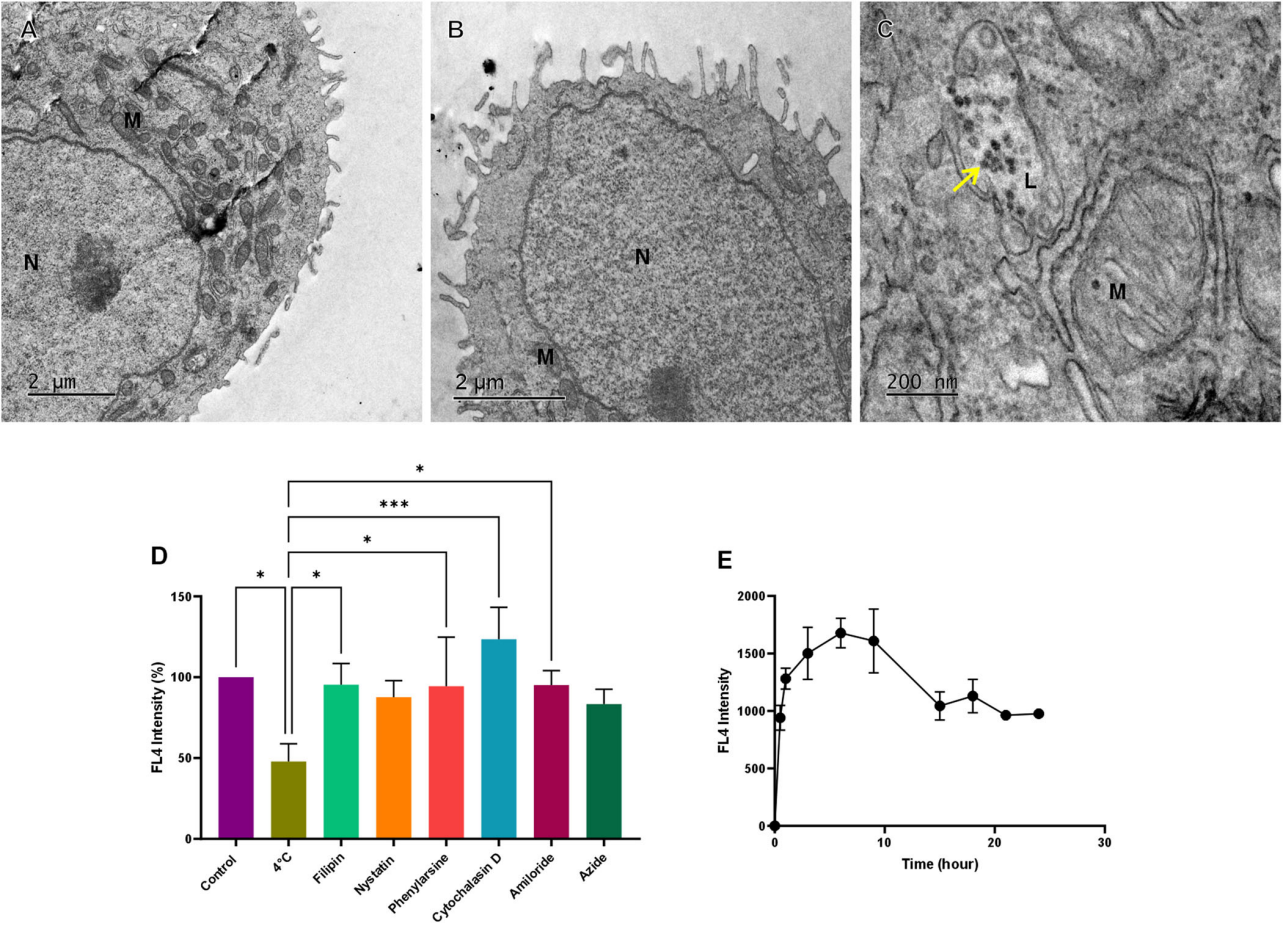
Internalization of SLN‐FTALO by AGS cells. The cells were treated with 30 mM of FTALO present in SLN‐FTALO for 3 h (A), 6 h (B–D), and in E, the exposure was up to 24 h. A, B, and C show micrographs obtained by transmission electron microscopy; the yellow arrow indicates internalized SLN‐FTALO. D represents the assay of blocking internalization pathways, statistics obtained *p* values of 0.0127 (control), 0.0259 (Filipin), 0.0271 (Amiloride), 0.0003 (Cytochalasin D), and 0,0299 (Phenylarsine) in comparison with 4°C treatment. E shows the variation of phthalocyanine fluorescence intensity in the cells over time. N stands for nucleus, M for mitochondria, and L for lysosomal vesicles.

The morphology during SLN‐DTX internalization was evaluated at 3 h (Figure 4A) and 6 h (Figure 4B,C), showing the cytoplasmic structures of endocytosis (Figure 4C). With the internalization pathway assay, endocytosis inhibitors are used to simulate the pathway used by SLN‐FTALO to reach the cytoplasm. Figure 4D shows the changes observed in the internalization of the nanostructures. Only inhibition by temperature at 4°C was able to reduce the fluorescence of the internalized marker, indicating passive internalization of SLN‐FTALO, presenting the profile (Figure 4E) with a peak in less than 10 h of treatment.

### 3.2 SLN‐DTX Treatment Alters AGS Cell Metabolism

**FIGURE 5:**
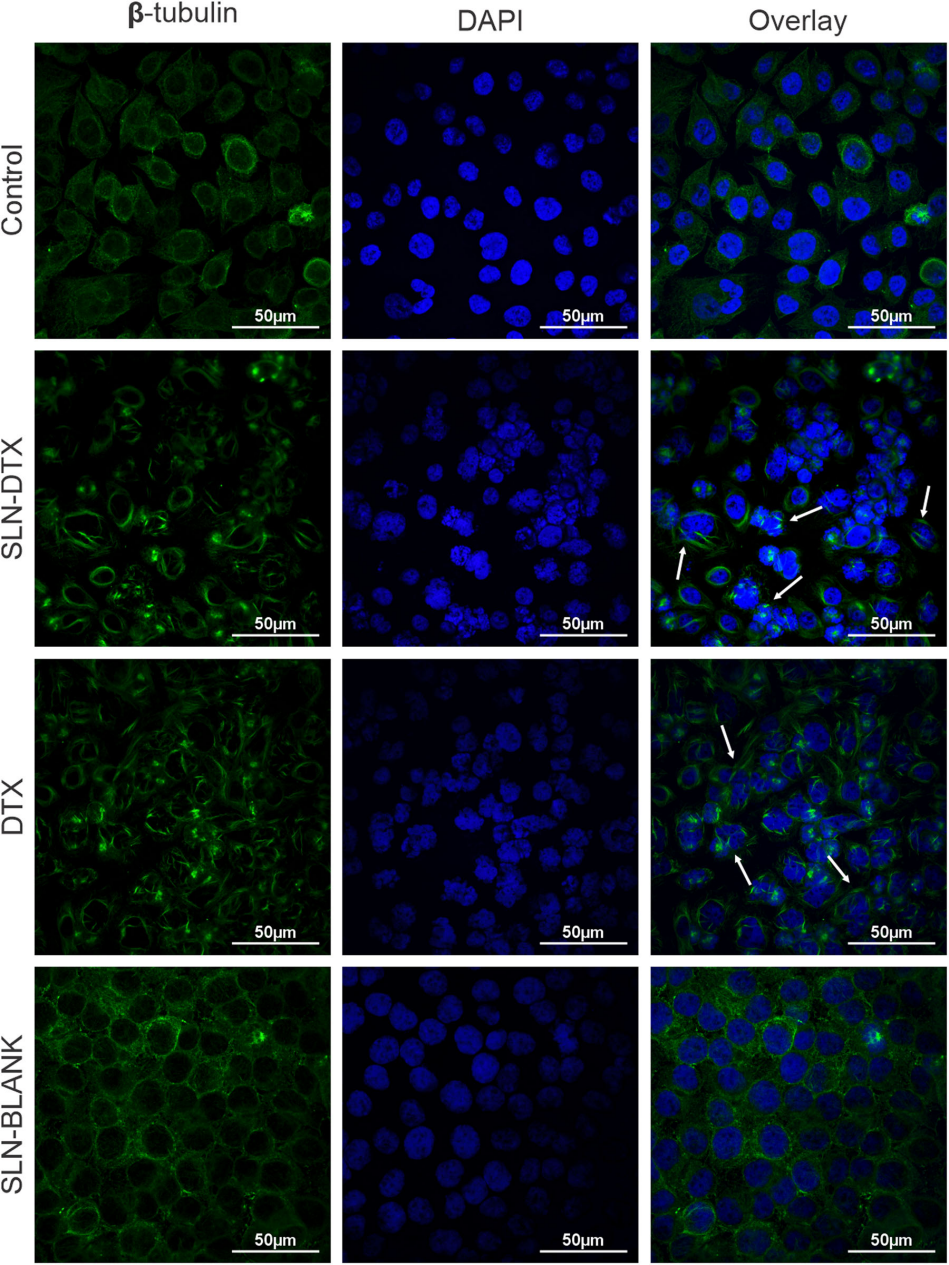
Evaluation of the cytoskeletal morphology of SLN‐DTX‐treated AGS cells by confocal laser scanning microscopy. β‐tubulin is represented in green, and the nuclei were stained with DAPI in blue; white arrows indicate cells with mitotic arrest after treatment.

Docetaxel causes the stabilization of microtubules in cells, inducing death after failure to complete the cell cycle. One of the observations of its activity is the presence of cells with a stabilized mitotic spindle and a nucleus in the process of division. For this purpose, fluorescent markers for β‐tubulin are used under confocal microscopy. In Figure 5, the white arrows indicate cells that present cytoskeletal proteins in spindle conformation, which are morphological changes caused by β‐tubulin stabilization by docetaxel treatment, inducing cell cycle arrest in the G2/M phase, leading to the death of cancer cells.

**FIGURE 6:**
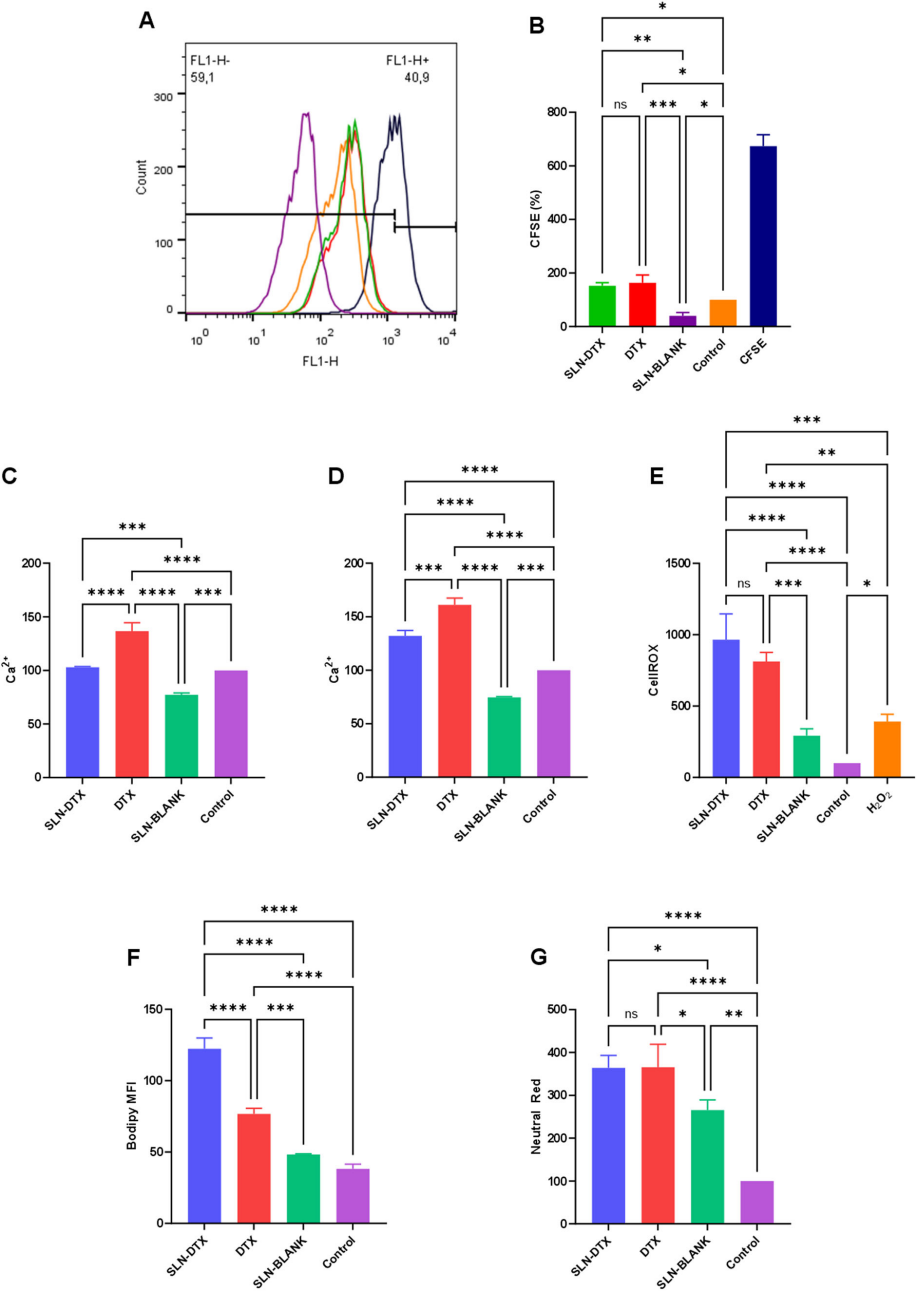
Modulation of AGS cell metabolism with SLN‐DTX treatment. A and B show the evaluation of proliferation by CFSE in flow cytometry. C and D show the release of intracellular calcium at 24 and 48 h, respectively. E shows the quantification of reactive oxygen species released after treatment. F quantifies lipid bodies and in G the neutral red labeling of lysosomal vesicles. B statistics obtained *p* values for SLN‐DTX compared to SLN‐BLANK (*p* = 0.0492), and Control (*p* = 0.0492). For C, statistics comparing SLN‐DTX to DTX and SLN‐BLANK resulted in *p* values of < 0.0001 and 0.0002, respectively. D resulted in *p* values of < 0.0001 (Control, and SLN‐BLANK) and 0.0001 when compared to SLN‐DTX. E statistics obtained *p* values of 0.0001 for H2O2, and < 0.0001 for SLN‐BLANK and Control compared to SLN‐DTX. Statistics in F presented *p* values of < 0.0001. SLN‐BLANK, and Control presented *p* values of 0.0248, and < 0.0001 respectively in comparison to SLN‐DTX. “ns” indicates nonsignificant statistical comparisons.

The metabolic alteration caused by SLN‐DTX treatment in AGS cells was evaluated through flow cytometry analysis. The CFSE proliferation assay was performed (Figure 6A,B), and SLN‐DTX showed a statistical difference in untreated cells, indicating lower proliferation after treatment.

Intracellular calcium (Figure 6C,D) significantly increased in AGS cells treated with SLN‐DTX, suggesting enhanced signaling potential for immunogenic cell death. Additionally, the production of reactive oxygen species (ROS) rose following treatment (Figure 6E), contributing to cellular damage, as evidenced by the increased lipid peroxidation (Figure 6F). Furthermore, Figure 6G demonstrates greater neutral red intensity, indicating enhanced lysosomal internalization in SLN‐DTX‐treated cells.

**FIGURE 7:**
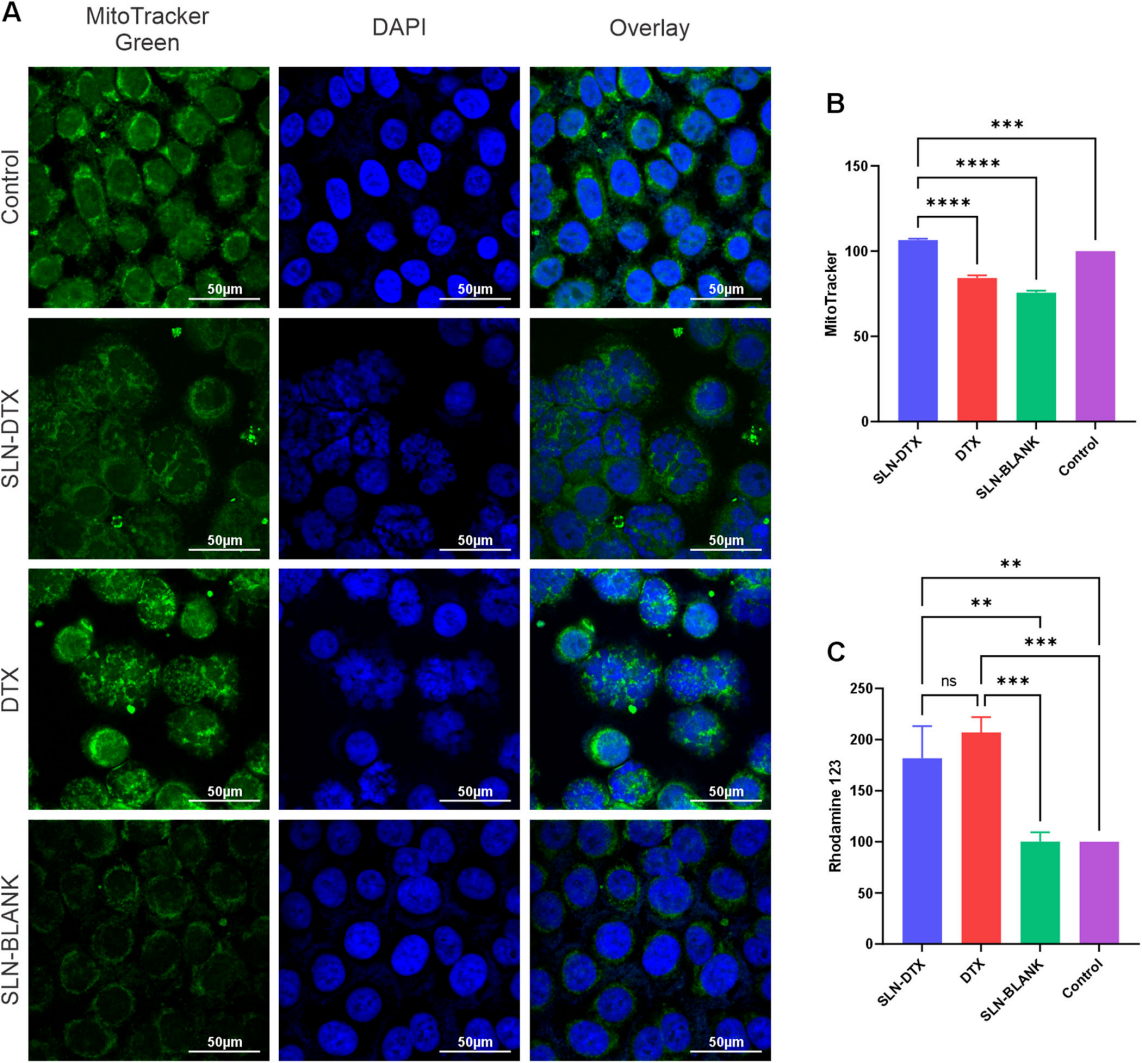
Evaluation of mitochondria by confocal microscopy and flow cytometry. (A) shows the fluorescence microscopy of AGS cells treated with SLN‐DTX where the mitochondria are labeled green with MitoTracker Green and the nuclei labeled with DAPI in blue; the quantification of mitochondrial fluorescence is represented in (B), *p* values of 0.0004 for control and < 0.0001 for DTX and SLN‐BLANK in comparison to SLN‐DTX; (C) shows the fluorescence of rhodamine 123, which also labels mitochondria but is dependent on the stability of their membrane polarization; *p* values for SLN‐DTX compared to SLN‐BLANK of 0.0025, and to Control of 0.0024. “ns” indicates nonsignificant statistical comparisons.

Mitochondrial alterations were assessed using MitoTracker Green (MTG) and Rhodamine 123 through confocal microscopy (Figure 7A) and flow cytometry (Figures B and C). SLN‐DTX‐treated cells exhibited increased accumulation of MTG in mitochondria, which is independent of mitochondrial membrane potential. In contrast, Rhodamine 123 staining indicated a loss of mitochondrial membrane potential due to depolarization [13]. Furthermore, the mitochondrial distribution was visibly altered in SLN‐DTX‐treated cells compared to the untreated control.

**FIGURE 8:**
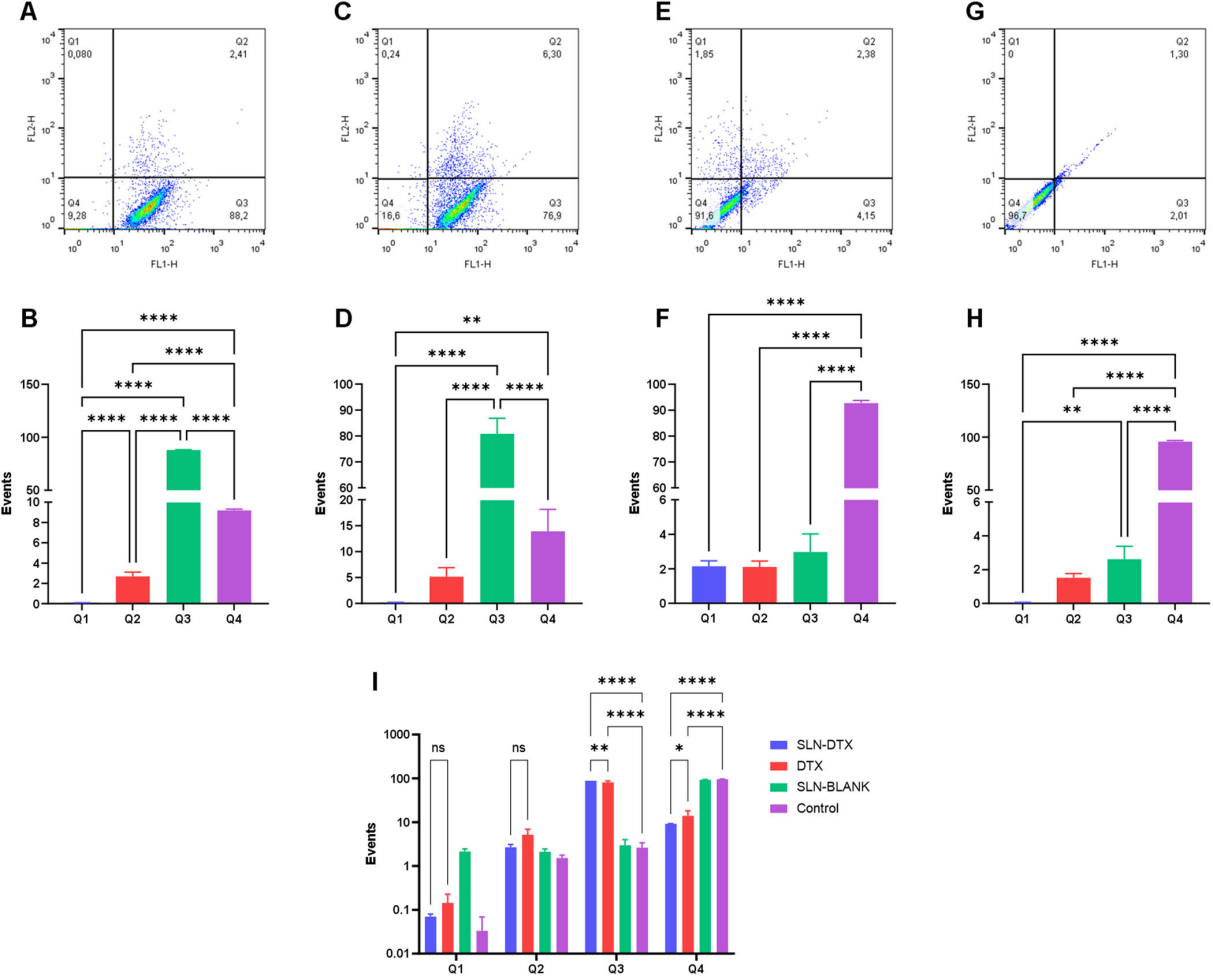
Cell death type assay with annexin V and propidium iodide performed in flow cytometry, showing the dot plot and quantification of events in each type of cell death for treatments with SLN‐DTX (A and B), DTX (C and D), BLANK‐SLN (E and F) and control (G and H). Quadrant Q1 represents cells positive for propidium iodide, signaling death by necrosis, Q2 presents cells positive for propidium iodide and annexin V, in Q3 are cells marked by annexin V, representing death by apoptosis, and in Q4 are viable cells. In I we have the comparison between groups for each quadrant and their significance. Statistics obtained *p* values of < 0.0001 represented by ****, in D, *p* = 0.0089 for **, in H, ** *p* = 0.0054, in I “ns” indicates nonsignificant, *p* = 0.0314 for * and *p* = 0.016 for **.

The annexin V and propidium iodide assay by flow cytometry is widely used to evaluate the type of cell death following treatment. Figure 8 shows the cell death profile after treatment with SLN DTX (A), with increased phosphatidylserine signaling on the outside of the cell, indicating cell death by apoptosis. Figure 8I shows the statistical comparison between the groups for the different types of cell death and controls, where a greater number of positive events for apoptosis and a smaller number for intact cells are presented.

### 3.3 SLN‐DTX Treatment Modulates the Immune Response in AGS Cells

**FIGURE 9:**
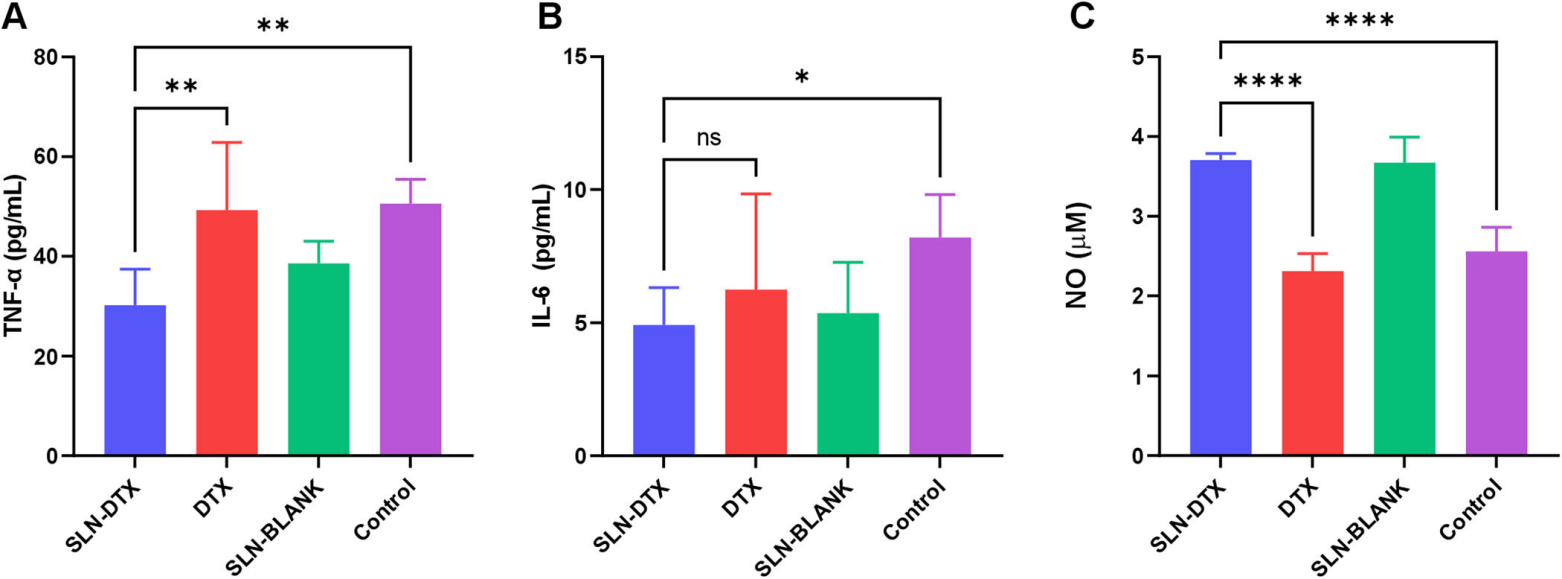
Quantification of cytokines and chemokines secreted by AGS cells treated with SLN‐DTX. (A) Tumor necrosis factor alpha, *p* = 0.0091 for DTX and = 0.0053 for Control in comparison to SLN‐DTX. (B) Interleukin 6, *p* = 0.0211. (C) Nitric Oxide, *p* < 0.0001 for ****, “ indicates non‐significant.

Following the evaluation of cytotoxic potential and metabolic changes, the levels of pro‐inflammatory cytokines TNF‐α, IL‐6, and nitric oxide were quantified in AGS cells treated with SLN‐DTX (Figure 9). A reduction in levels of TNF‐α and IL‐6, cytokines known to drive tumor progression, inflammation, and metastasis formation, was observed. TNF‐α is a key marker for assessing the prognosis of gastric cancer patients, while IL‐6 enhances AGS cell invasiveness, contributing to metastatic progression. Conversely, nitric oxide levels rose in SLN‐DTX‐treated cells, suggesting enhanced antitumor activity, inhibition of cell proliferation, and increased oxidative stress.

## 4 Discussion

Optimizing chemotherapy treatments for gastric cancer is crucial to improving prognosis, increasing survival rates, and enhancing treatment efficacy. The use of SLN‐DTX resulted in a significant reduction in AGS cell viability (Figure 1), with an IC50 value comparable to that reported by da Rocha et al. 2020 [12] in 4T1 murine mammary adenocarcinoma cells, further confirming the cytotoxicity potential of SLN‐DTX treatment. The combination of chemotherapeutics with solid lipid nanoparticles has introduced several positive advancements in cancer therapy. For instance, in other studies conducted by the group, the incorporation of doxorubicin into SLN‐BLANK (referred to as SLNDox) successfully prevented bone loss associated with chemotherapy and breast cancer in mice [14].

Morphological changes in the treated cells were consistent with cytotoxicity, including a significant reduction in cell density on the surface (Figure 2D). Additionally, alterations in cell morphology were observed, characterized by decreased cytoplasmic extensions and a reduced number of cells exhibiting a more adherent phenotype (Figure 2E,F), as described by Hou et al. 2021 [15]. The DTX present in SLN‐DTX promotes cell death through the stabilization of microtubules, altering the morphology of the cell by modifying the organization of the cytoskeleton. Paiva et al. 2022 [16] and da Rocha et al. 2020 [12] observed similar morphological changes in MDA‐MB‐231 and 4T1 cells treated with SLN‐DTX.

The cytotoxic effects of SLN‐DTX on gastric cancer cells are evident in changes to their internal morphology. Zhang et al, 2018 [17] reported that damaged AGS cells exhibited enhanced lysosomal activity. In Figure 3C,D the presence of lysosomal vesicles is visible, and in Figure 6G, an increase in lysosomal activity is noted in AGS cells treated with SLN‐DTX. The increase in lysosomal vesicles following treatment may be associated with the internalization of nanostructures, their binding to the cell membrane, and subsequent endocytosis (Figure 4). The cytotoxicity induced by SLN‐DTX led to reduced wound‐healing capacity in treated cells compared to the control (Supporting Information S1: Figure 1), consistent with the cytoskeleton alterations caused by the treatment. Yang et al., 2020 [18] similarly reported impaired wound healing in AGS cells following treatment.

As previously observed, the cytotoxic effects of SLN‐DTX disrupt cytoskeleton organization, causing alterations in the mitotic spindle and dividing nuclei (Figure 5). These disruptions result in mitotic failure, cell cycle arrest, checkpoint errors, and ultimately cell death, consistent with docetaxel's mechanism of action.

Flow cytometry analysis (Figure 8) confirmed apoptosis as the primary mode of cell death in SLN‐DTX‐treated cells. Figure 6C,D shows an increase in intracellular calcium release, suggesting that apoptosis‐associated calcium externalization may contribute to immunogenic cell death. Additionally, Figure 6A,B demonstrates reduced cell proliferation due to cell death, as initially described in Figure 1.

The cytotoxicity process also leads to elevated reactive oxygen species (ROS), lipid peroxidation (Figure 6E,F), and increased nitric oxide release (Figure 9C). Yao et al. 2015 [19] defined nitric oxide release as a mechanism for inhibiting cell proliferation in gastric cancer, highlighting its role in modulating the tumor microenvironment′s immunological profile.

The increased mitochondrial staining observed in Figure 7 suggests that treatment significantly alters mitochondrial function, which plays a crucial role in the therapeutic process of cancer cell elimination. Disrupted mitochondrial function can induce a pro‐oxidant profile, enhancing the efficacy of antitumor therapies. In line with this, the pro‐oxidant changes induced by SLN‐DTX treatment led to alterations in the levels of TNF‐α and IL‐6 (Figure 9A,B). Roșu et al. 2022 [20] described TNF‐α as used as a biosensor for the progression of gastric cancer, promoting an inflammatory profile favorable for tumor growth and success. Treatment with SLN‐DTX reduced TNF‐α levels, playing a crucial role in improving the prognosis. In addition to TNF‐α, IL‐6 levels were also significantly reduced. Ashizawa et al. 2005 [21] highlighted the clinical significance of IL‐6 in gastric cancer prognosis, noting that IL‐6 promotes invasiveness and metastasis progression. Its reduction, therefore, has a positive impact on patient outcomes, further supporting the potential of SLN‐DTX as an effective therapeutic strategy.

Nanostructured docetaxel (SLN‐DTX) has advantages over the commercial form of the drug by reducing the dose required to induce death in gastric cancer cells (Figure 1), and the drug′s action reduced the presence of filopodia, cytoplasmic structures responsible for migration and invasiveness in cancer cells (Figures 2 and 3). SLN‐DTX is composed of low‐toxicity lipids, and it presents an appropriate size [12] for the optimization of bioavailability and for the pharmacokinetic and pharmacodynamic optimization proposed by nanobiotechnology. It thus boosts the therapeutic potential of docetaxel by combining the advantages of nanobiotechnology with the drug′s mechanisms of action, with an emphasis on greater efficacy and reduction of adverse effects.

## 5 Conclusion

Effective treatment of gastric cancer is crucial for significantly reducing global mortality associated with the disease. Treatment with docetaxel associated with solid lipid nanoparticles induced cytotoxicity in AGS cells, resulting in morphological alterations in the cytoskeleton, changes in the distribution and organization of organelles, modulation of the cell death profile, and significant effects on cellular metabolism. The treatment was also able to increase the presence of reactive oxygen species and modulate the inflammatory profile, reducing pro‐tumor responses. Thus, SLN‐DTX emerges as a promising therapeutic strategy in gastric cancer treatment, offering the potential to minimize treatment‐related side effects while enhancing overall prognosis.

Furthermore, combining docetaxel with solid lipid nanoparticles offers an innovative therapeutic approach by enhancing treatment efficacy and minimizing the common side effects of conventional therapies. These results suggest that using SLN‐DTX could represent a significant advancement in gastric cancer treatment, not only due to its direct antitumoral action but also for its positive impact on the modulation of metabolic and inflammatory pathways. Although further clinical studies are needed to validate these findings in human models, the experimental data obtained thus far indicate that SLN‐DTX therapy could be an important step towards more effective and less toxic treatments for gastric cancer patients, offering improved quality of life and long‐term survival.

## Author Contributions

L.V.‐C. and M.A.R. conceived the research, designed the experiments, and wrote the manuscript. S.N.B. conceived the research, acquired the funding, and supervised the study. M.A.R. carried out the preparation and characterization of SLN‐BLANK, SLN‐DTX and participated in the editions of the figures. G.S.S.S.T. performed transmission and scanning electron microscopies. K.G.M. carried out the acquisition of confocal fluorescence microscopy images. S, IG, and K.G.M. performed the experiments and acquired data on cytokines, nitric oxide, and lipid body biogenesis. All authors read and approved the final manuscript.

## Supporting information

Supplementary_Material_25_July.

## Data Availability

The data that support the findings in this study are available from the corresponding author upon reasonable request.
