# Supplementary material for: Enhanced Cytotoxic Effects of Docetaxel‐Loaded Solid Lipid Nanoparticles (SLN‐DTX) on Gastric Adenocarcinoma In Vitro
Source: J Biochem Mol Toxicol. 2025 Aug 21;39(9):e70456. doi: 10.1002/jbt.70456 (PMC12369453; doi:10.1002/jbt.70456)
Supplement: Supplementary file 1 — Supplementary_Material_25_July. [file JBT-39-e70456-s001.docx]

**Enhanced Cytotoxic Effects of Docetaxel-Loaded Solid Lipid Nanoparticles (SLN-DTX) on Gastric Adenocarcinoma in vitro**

Laís Vaz-Costa¹^,2^, Marina Arantes Radicchi¹^,2^, Caterynne Melo Kauffmann^3^, Guilherme Sirimarco de Souza Silveira Tonelli^2^, Igor Oliveira Santos^4,^ Kelly Grace Magalhães^4,^ Sônia Nair Báo^2^*

*Correspondence: [snbao2009@gmail.com](mailto:snbao2009@gmail.com)

^1^Graduate Program in Biological Sciences (Molecular Biology), Department of Cell Biology, Institute of Biological Sciences, University of Brasilia, Brasília, Brazil

^2^Laboratory of Microscopy and Microanalysis, Institute of Biological Sciences, University of Brasilia, Brasília, Brazil

^3^Laboratory of Phytopathology, Institute of Biological Sciences, University of Brasília, Brasília (DF) Brazil

^4^ Laboratory of Immunology and Inflammation, Department of Cell Biology, University of Brasilia, Brasília, DF, Brazil

**Supplementary material**

**
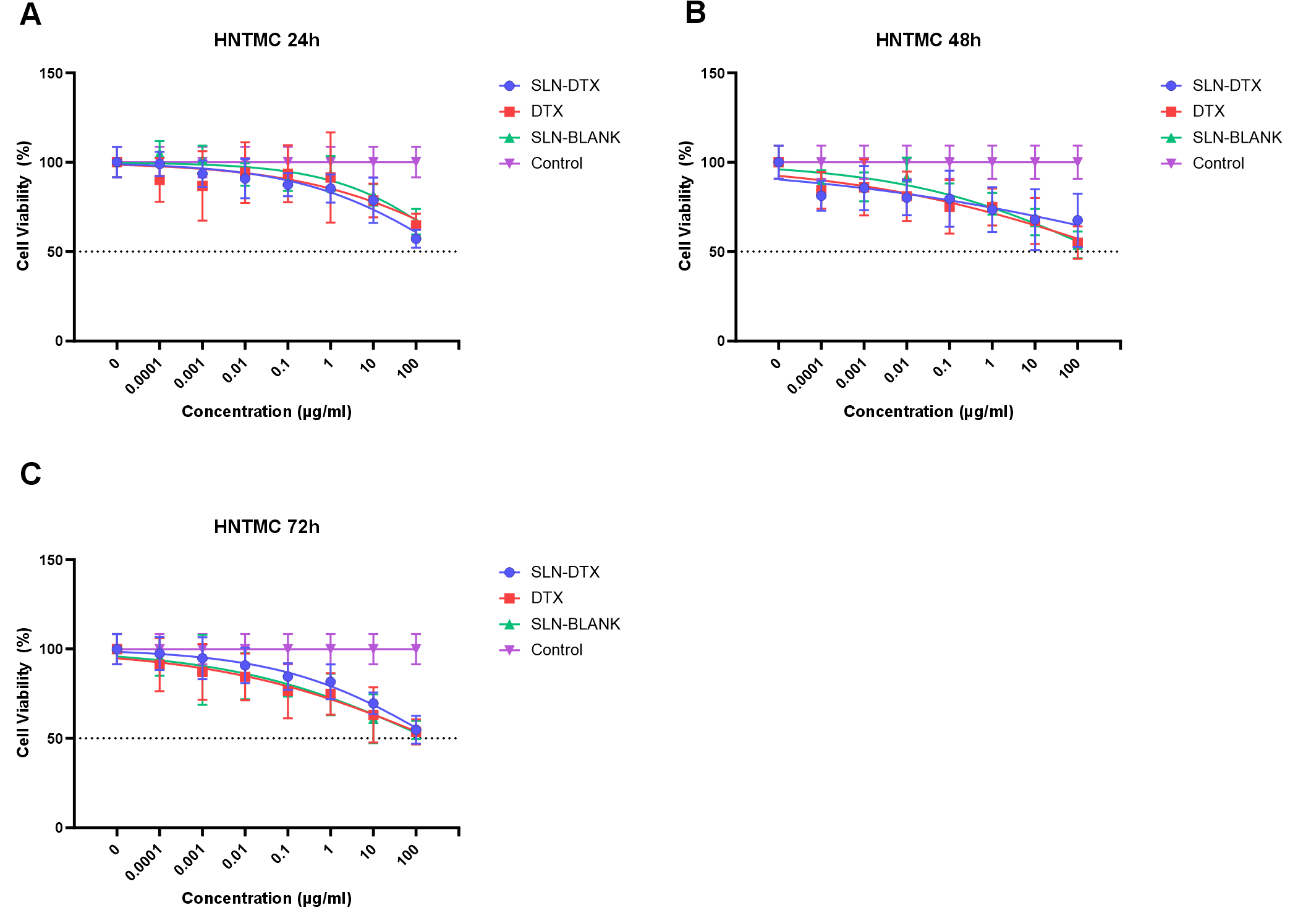
**

*Figure S1 – In vitro viability assay by MTT. Human fibroblast cells (HNTMC) were treated with increasing concentrations of SLN-DTX for up to 72 hours (A-C). The IC50 obtained were higher than 500 µg/mL for SLN-DTX treatments.*

**
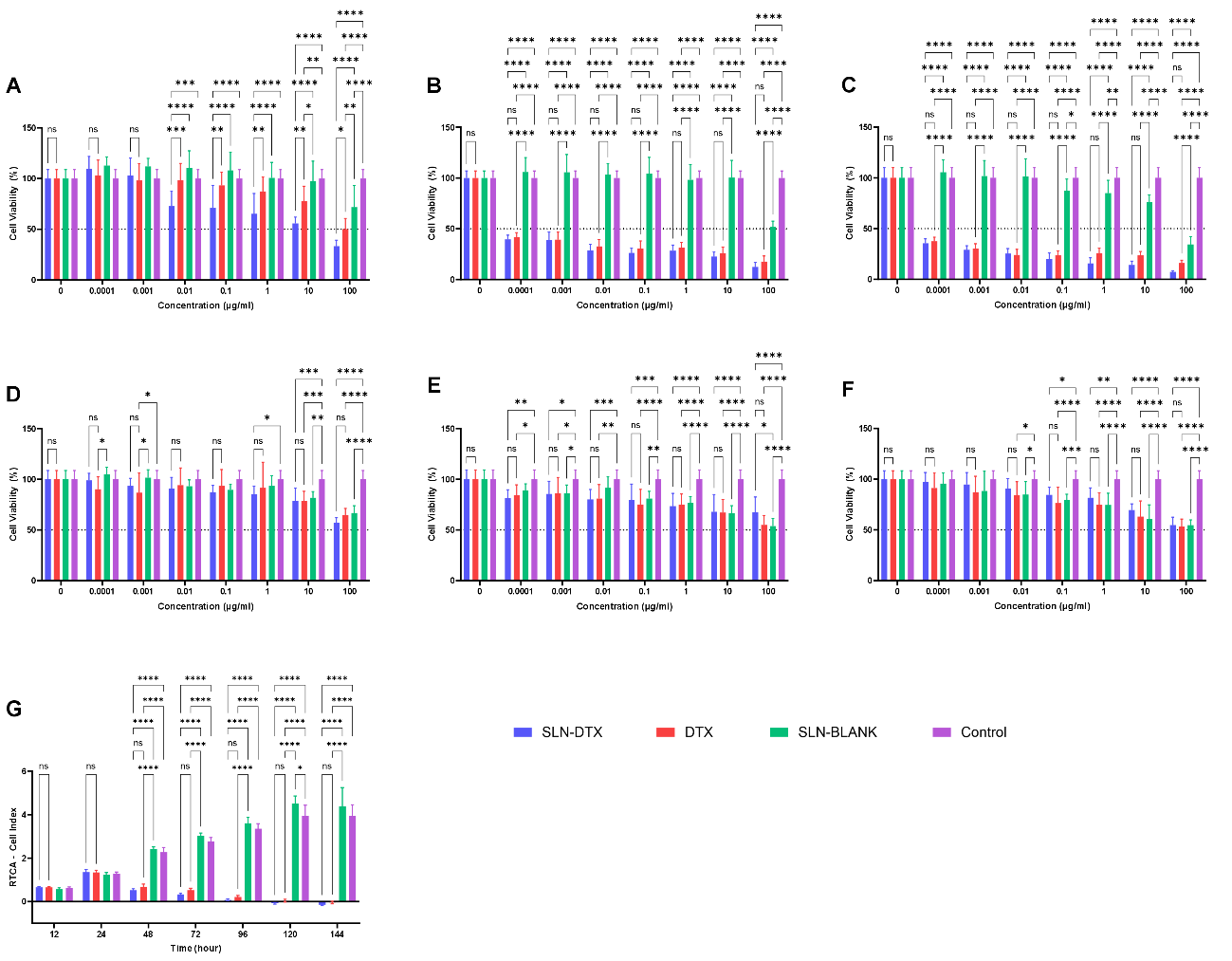
**

*Figure S2 – Statistical analysis of MTT and RTCA viability assays. A-C represent the cell viability of AGS after treatments of 24, 48 and 72h respectively. For the HNTMC cell line, the treatments at 24h (D), 48h (E) and 72h (F) are represented. In G the statistical comparison between groups for selected time points related to the RTCA experiment.*


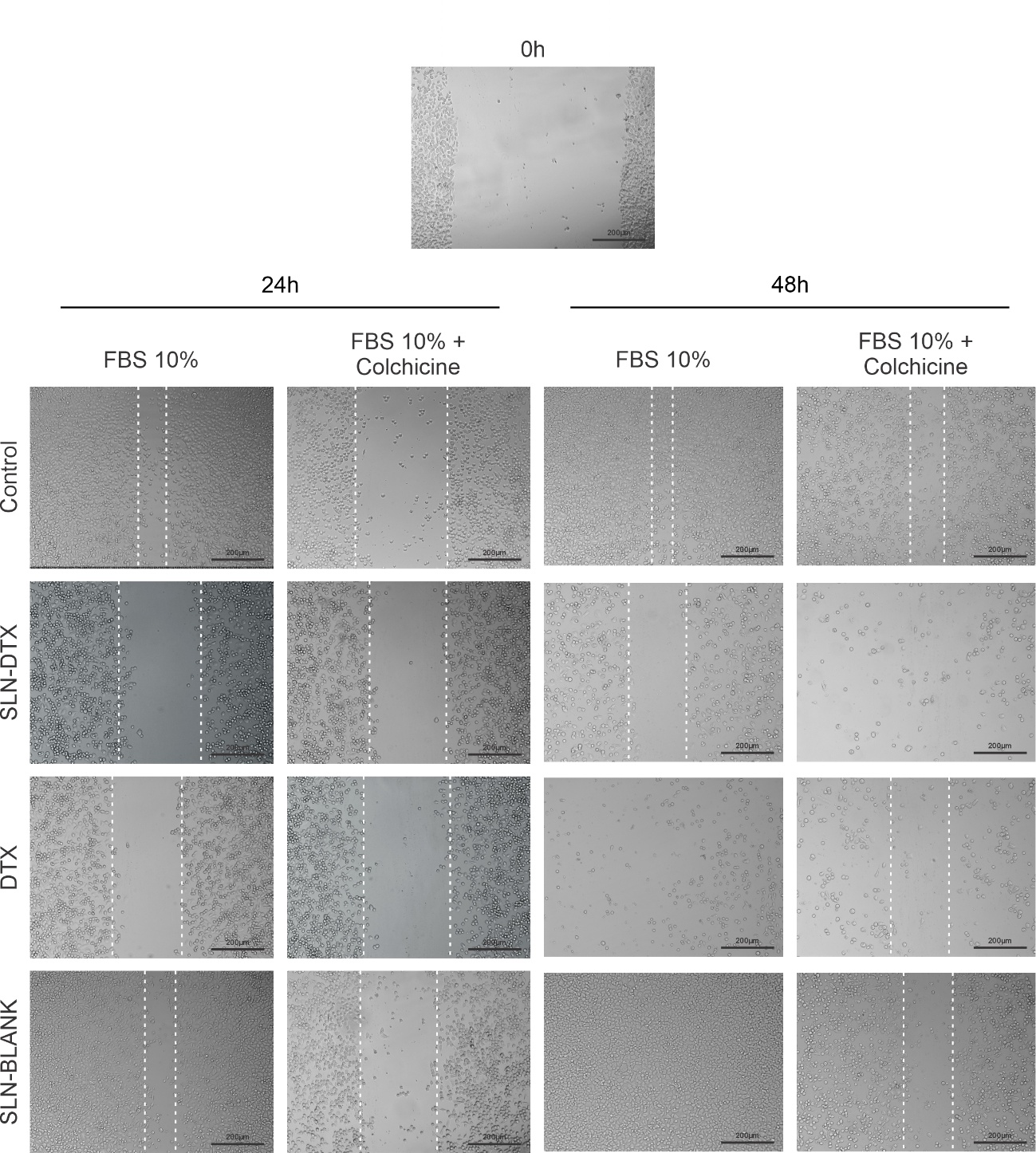


*Figure S3 – Plated SLN-DTX-treated AGS were wounded with the micropipette tip, wound healing was evaluated after 24 and 48h, and colchicine was used as proliferation control. Dotted lines indicate wound closure.*
